# SGLT2-inhibition and myocardial infarction size in patients with type 2 diabetes mellitus– Insights from an acute cardiovascular care center

**DOI:** 10.1186/s12872-025-04981-5

**Published:** 2025-08-02

**Authors:** Istvan Bojti, Felicitas Bojti, Tau Hartikainen, Philipp Breibart, Nikolaus Löffelhardt, Christian Valina, Kilian Franke, Klaus Kaier, Dennis Wolf, Dirk Westermann, Christoph B. Olivier

**Affiliations:** 1 Department of Cardiology and Angiology, University Heart Center Freiburg - Bad Krozingen, Faculty of Medicine, University of Freiburg, Hugstetter Str. 55, Freiburg, 79106 Germany; 2 Institute of Medical Biometry and Statistics, Faculty of Medicine and Medical Center, University of Freiburg, Freiburg, Germany

**Keywords:** SGLT2-inhibitors, Infarct size, Acute myocardial infarction, Diabetes mellitus.

## Abstract

**Aims:**

This study aimed to assess the association between myocardial infarction (MI) size and clinical outcome with ongoing Sodium-glucose-cotransporter-2 inhibitor (SGLT2-i) use.

**Methods and results:**

In this retrospective single-center cohort study 681 patients with MI and diabetes mellitus type 2 (T2DM) treated with percutaneous coronary intervention (PCI) between November 2015 and December 2023 were included. 105 patients received ongoing SGLT2-i therapy and 576 were taking other glucose-lowering medication at the time of admission. The primary outcome was the size of MI. MI size was determined by the peak high-sensitive troponinT (hs-TnT x ULN [upper limit of normal]) normalized on the endangered myocardial area (EMA). No significant statistical differences were observed in hs-TnT values (hsTnT x ULN: 55 [13–174] vs. 68 [22–182]; *p* = 0.36) and EMA (41 [29–59] vs. 35 [24–59] %; *p* = 0.24) between patients with and without SGLT2-i therapy. After augmented inverse-probability weighted analyses, ongoing SGLT2-i therapy was not significantly associated with a reduced MI size (difference between means hsTnT x ULN/EMA [1/%]: − 0.24 [95% confidence interval, − 2.95 to 2.48]; *p* = 0.54). Secondary outcomes were in-hospital major adverse events and length of intensive care unit (ICU) treatment. SGLT2-i use was not associated with fewer in-hospital adverse events (3.81, vs. 4.17% [95% CI, − 2.95 to 2.48], *p* = 0.94) or with fewer days on ICU (1 [1–1] vs. 1 [1–2] days, *p* = 0.78).

**Conclusion:**

In this retrospective cohort study of T2DM patients presenting with MI, prior prescription of SGLT2-i was not associated with reduced MI size or fewer adverse events during hospitalization for MI treated with PCI.

**Trial registration number and date:**

DRKS00032432 (Registration Date: 07 August 2023).

**Supplementary Information:**

The online version contains supplementary material available at 10.1186/s12872-025-04981-5.

## Introduction

Sodium-glucose-cotransporter-2 inhibitors (SGLT2-i) reduce insulin resistance, body weight, and blood pressure through increasing urinary glucose secretion in the proximal tubule of the kidney [1] and are also cardioprotective [2]. In patients with type 2 diabetes mellitus and high cardiovascular risk, SGLT2-i reduce cardiovascular death and heart failure-related hospitalizations [3] but their effects on myocardial infarction are less clear.

In prospective and retrospective clinical studies, the early initiation of an SGLT2-i after myocardial infarction (MI) was associated with a reduced risk of adverse cardiovascular events, lower mortality and improved echocardiographic parameters [4, 5]. However, in a recent, large-scale, international, prospective and randomized trial early SGLT2-i therapy after MI did not significantly reduce the risk of a first hospitalization for heart failure or death compared with placebo [6, 7]. None of the above mentioned studies considered the endangered myocardium area (EMA) as a potential confounding factor. EMA is a valuable tool for an early prediction of the myocardial infarct size [8] and predicts all-cause mortality and hospitalization for heart failure within 1 year [9].

Measurement of the infarct size is challenging and if there is no cardiac magnetic resonance imaging as gold standard available, the infarct size can only be estimated. Fortunately the correlation between cardiac troponins and infarct size proved to be strong enough to be used for this estimation [10].

Preclinical and clinical data suggest a protective effect even if the therapy was started before an ischemia/reperfusion injury [11–13]. Thus, SGLT2-i therapy may protect from adverse cardiovascular events, more specifically it reduces the size of an acute MI, and also reduces the risk of heart failure and improves prognosis. This study aimed to assess whether an ongoing SGLT2-i therapy in diabetic patients is associated with a reduced size of MI and secondary outcomes, such as in-hospital adverse events and ICU stay.

## Methods

### Study design

For this retrospective single-centre cohort study, patients who underwent percutaneous coronary intervention (PCI) for myocardial infarction (MI) and had type 2 diabetes mellitus (T2DM) between November 2015 and November 2023 were screened from a German tertiary care centre’s archive. The protocol and amendments were approved by the local ethics committee, privacy and security offices, and the institutional review board (Ethik-Kommission Albert-Ludwigs-Universität Freiburg, 21-1667). The study was performed in accordance with the ethical standards laid down in the 1964 Declaration of Helsinki and its later amendments. The study was registered on the “German Clinical Trials Register” (DRKS00032432).

### Study population

Patients with both acute ST-segment elevation (STEMI) and non-ST-segment elevation myocardial infarction (NSTEMI) accompanied by DM undergoing PCI over the age of 18 years were screened. The archival research included ICD-10 codes I21 and E11 simultaneously and included patients only if they received a PCI. Therapy and the definition of STEMI/NSTEMI followed the then current guidelines of the European Society of Cardiology [14, 15]. Diabetic patients were identified according to the ICD-10 code E11. Patients were grouped according to the medication being taken at admission: SGLT2-i users or no-SGLT2-i users. Exclusion criteria were the following: absence of any glucose-lowering medication, acute MI directly after coronary artery bypass grafting (Type 5 MI), peri-interventional MI (Type 4a MI), a glomerular filtration rate (GFR) under 25 ml/min/1,73 m² – a contraindication for the prescription of SGLT2-i inhibitors – and/or a haemoglobin value under 8 g/dl at admission. After identification of the suitable patients for this study a licensed MD reviewed the patients´ charts. Quality control was performed by a board-certified internist and discordances were resolved by a board-certified cardiologist.

### Infarct size quantification

According to the in-house protocol high-sensitivity troponin T (hs-TnT; Roche, Switzerland) values were available at 8, 16, 24, and 48 h after PCI – or until death if it occurred earlier. The highest value hs-TnT_max_ was used to estimate the peak, which correlates best with the infarct size among cardiac biomarkers [16]. Values are provided as times the upper limit of normal (ULN).

### Laboratory parameters

The measured laboratory parameters are listed in Supplementary Table 1. If not specified otherwise, parameters were included from the time point of admission.

### Area-at-risk determination

All patients underwent a coronary angiogram followed by a PCI. Based on the primary coronary angiogram, two independent investigators determined the coronary dominance and the length of the left anterior descending artery (LAD). In case of discrepancies, a joint determination of the questioned parameters followed. The culprit lesion was considered as the treated lesions during primary coronary care. The number of ischemia-affected left ventricular (LV) segments was calculated based on the coronary dominance, the length of the LAD and the localisation of the culprit lesion(s) according to the holistic coronary care algorithm [17] using the standardized 17-segment polar map of the myocardium [18]. The area at risk was expressed as percent (number of ischemia-affected LV segments/17*100). The hs-TnT_max_ level was normalized to the area at risk to determine the size of MI.

### Clinical outcome

Killip classification at presentation, the duration of intensive care unit (ICU) stay and in-hospital treatment were assessed by chart review [19]. The left ventricular ejection fraction (LVEF) was documented from the post-MI transthoracic echocardiogram. A screening for in-hospital death or stroke as major adverse events was also performed based on chart review.

### Statistics

Continuous variables were described using median and interquartile range (IQR). Unadjusted between-groups comparisons based on Mann–Whitney tests in the variable were not normally distributed. In case of a log-normal distribution, values were transformed. Normal or log-normal distributed variables (Kolmogorov–Smirnov test) were compared using a two-sided t-test. Categorical variables were described using absolute and relative frequencies; unadjusted between-groups comparisons were made using Fisher’s exact test (2 × 2 contingency tables) or Chi square test (2 × 3 contingency tables).

The propensity score method was used to compare treatment groups. We applied lasso (least absolute shrinkage and selection operator) for variable selection. Covariates defined by the method are: age, sex, DDP4-therapy, Insulin-therapy, diuretics-therapy, MRA-therapy, ARB-therapy, BB-therapy, CCB-therapy, ticagrelor-therapy, coronary heart disease, heart failure, smoking, atrial fibrillation and peripheral artery disease. In detail, an adaptive lasso was applied in order to avoid the over-selection of covariates with zero coefficients. Apart from these variables, HbA1c and glucose level at admission was defined as clinically relevant from a clinical perspective and therefore included in the final model for further analysis. The Stata´ command telasso was used, which combines adaptive lasso with the propensity score based augmented inverse-probability weighting (AIPW)] technique. AIPW is a combination of 2 models: the inverse probability weighting in the treatment model and the regression adjustment in the outcome model. We expressed the effects of SGLT2-i therapy in terms of the average treatment effect (ATE), defined as the mean difference (or risk difference) between patients who were under SGLT2-i therapy or not. The balance of the covariates was checked by the inspection of balancing statistics (the standardized mean difference of covariate distribution between treatment groups) and the application of overidentification tests (Supplementary Fig. 1).

Power calculation: as a skewed distribution of the maximal normalized hs-TnT is expected, a non-parametric two-sided Mann–Whitney U-test at a significance level of 5% will be used. Given a C-index of 70%, a sample size of 133 observations per group is necessary to achieve an adequate power of 80% to reject null hypothesis.

Three-year survival was analysed using pseudo-observations and linear regression with application of the method by Overgaard, Anderson and Parmer [20].

## Results

### Patient population

**Fig. 1:**
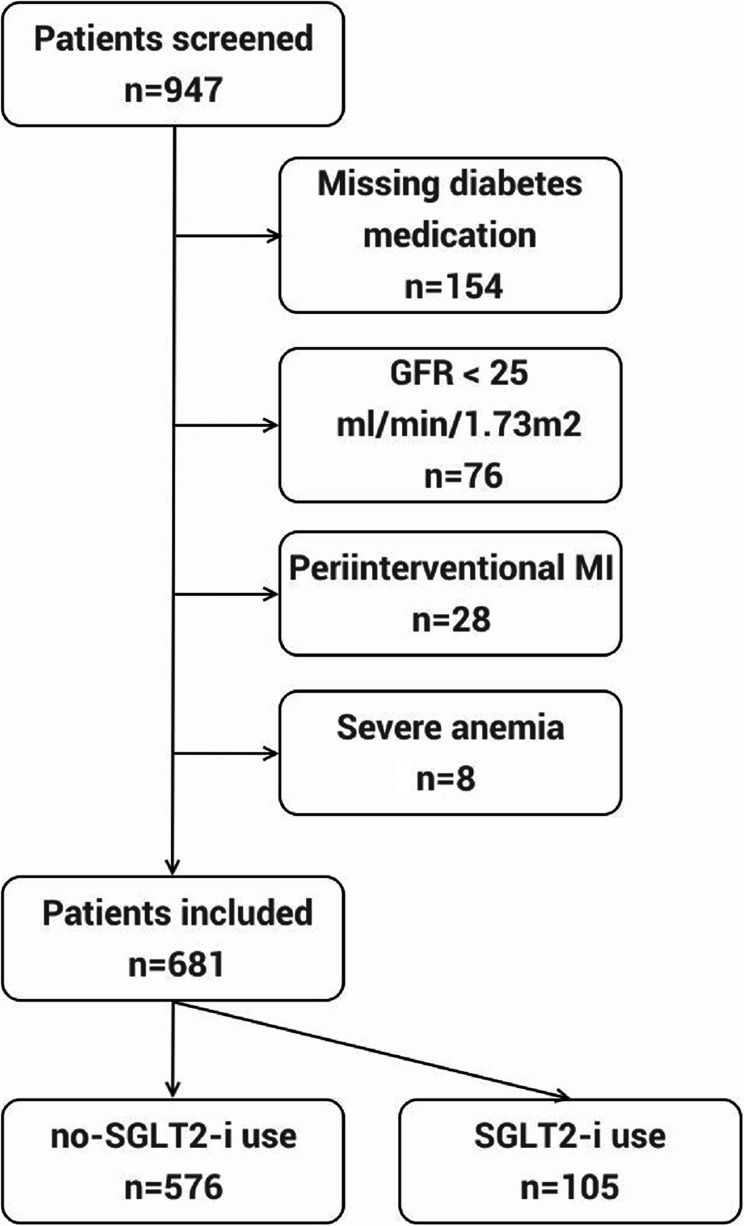
Screening flow chart

In total, 947 patients were treated with PCI due to acute MI who had simultaneous DM during study period. After exclusion of patients with missing diabetes medication (*n* = 154), severe renal insufficiency (GFR under 25 ml/min/1,73 m², *n* = 76), severe anemia (hemoglobin under 8 g/dl, *n* = 8) or periinterventional MI (*n* = 28), 681 patients qualified for analysis and were grouped according to glucose lowering medication: 105 patients in the SGLT2-i and 576 in the no-SGLT2-i group (Fig. 1).

Patients in the SGLT2-i group were significantly younger (median age [years]: 67 [interquartile range, IQR 60–75] vs. 72 [64–80], *p* < 0.001) and male sex was more frequent (80.0% vs. 69.1%, *p* = 0.026) compared with the no-SGLT2-i group. There was no significant difference regarding cardiovascular risk factors: family history, arterial hypertension, hyperlipidemia or smoking. Compared with the no-SGLT2-i group, prior coronary heart disease (57.1% vs. 42.7%, *p* = 0.008) or systolic heart failure (20% vs. 10.2%, *p* = 0.008) were more common in the SGLT2-i group. The use of a beta-blocker was significantly more frequent in the SGLT2-i group compared with the no-SGLT2-i (61% vs. 48.8%), *p* = 0.026). No significant difference was observed in the use of other frequent cardiovascular medication between the two study groups. Baseline characteristics of both groups are shown in Table 1.

**Table 1:** Baseline characteristics

	SGLT2-i use				
	Yes (*N* = 105)	No (*n* = 576)			*p*-value
**General**					
Age (years) *	67 (60–74.5)	72 (64–79.8)			< **0.001**
Male sex n (%)*	84 (80)	398 (69.1)			**0.026**
BMI	29 (26–32.5)	28 (26–32)			0.40
**Cardiovascular risk factors**					
Family history n (%)	31 (29.5)	132 (22.9)		0.17	
Arterial hypertension n (%)	91 (86.7)	496 (86.1)		0.99	
Hyperlipidemia n (%)	82 (78.1)	436 (75.7)		0.71	
Active smoking n (%)*	18 (17.1)	105 (18.2)		0.89	
**Medical history**					
Coronary heart disease n (%)*	60 (57.1)	246 (42.7)		**0.008**	
CABG n (%)	11 (12.9)	45 (8.4)		0.22	
Prior infarction n (%)	34 (32.4)	147 (25.5)		0.15	
Multiple diseased vessels n (%)	48 (45.7)	206 (35.8)		0.06	
Atrial fibrillation n (%)*	16 (15.2)	89 (15.5)		0.99	
Heart failure n (%)*	21 (20)	59 (10.2)		**0.008**	
Peripheral artery disease n (%)*	17 (16.2)	111 (19.3)		0.50	
Cerebral artery disease n (%)	12 (11.4)	88 (15.3)		0.37	
**Medication**					
Metformin n (%)	77 (73.3)	401 (69.6)		0.49	
DDP4i n (%)*	25 (23.8)	170 (29.5)		0.23	
Sulfonylurea n (%)	1 (1)	42 (7.3)		**0.008**	
Insulin n (%)*	49 (46.7)	231 (40.1)		0.24	
GLP-1a n (%)	2 (1.9)	7 (1.2)		0.64	
Diuretics n (%)*	38 (36.2)	233 (40.5)		0.45	
MRA (%)*	13 (12.4)	39 (6.8)		0.07	
CCB*	37 (35.2)	188 (32.6)		0.65	
ARNI/ACEi/ARB n (%)*	74 (70.5)	383 (66.5)		0.49	
Beta blockers n (%)*	64 (61)	281 (48.8)		**0.026**	

Continuous variables were described using median and interquartile range (IQR), categorical variables were described using n (%). *p*-values are from Mann–Whitney tests (continuous variables) or Fisher’s exact test (categorical variables). Revascularisation in the medical history is defined as previous coronary artery bypass grafting and/or percutaneous coronary intervention. Heart failure in the medical history comprises HFpEF, HFmrEF and HFpEF

Bold font indicates statistical significance

*Abbreviations*: *ACEI* angiotensin-converting enzyme inhibitor, *ARB* angiotensin receptor blocker, *ARNI* angiotensin receptor/neprilysin inhibitor, *BMI* body mass index, *CCB* Ca2 + channel blocker; DDP4i; Dipeptidyl peptidase-4 inhibitor, *GLP-1a* glucagon-like peptide-1 agonist *HFr/mr/pEF* heart failure with reduced/mid-range/preserved ejection fraction, *IQR* interquartile range, *MRA* mineralocorticoid receptor antagonist, *SGLT2-i* Sodium-glucose Cotransporter-2 Inhibitors

* indicates parameters used in the covariate balancing

Supplementary Table 1 presents the unweighted laboratory findings at admission. Laboratory findings showed a significantly lower but normal hemoglobin value in the no-SGLT2-i group compared to the SGLT2-i group (14.6 [13.4–15.6] vs. 13.3 [12–14.6] d/dl, *p* < 0.001). There was a mild renal insufficiency in both groups with a lower glomerular filtration rate (GFR) in the no-SGLT2-i group compared to the SGLT2-i group (78 [57.5–94] vs. 69.8 [50–88.2] ml/min/1.73 m², *p* = 0.017). Hematocrit values were in the normal range in both groups although significantly higher in the SGLT2-i group compared to the no SGLT2-i group (43.6 [40–45.8] vs. 39.5 [35.8–42.8], *p* < 0.001). The glycated hemoglobin (HbA1c) levels at admission were found to be significantly higher in the SGLT2-i group compared to the no-SGLT2-i group (7.6 [6.9–8.825] vs. 7.2 [6.6-8] %, *p* < 0.001). There was no statistically significant difference regarding the serum glucose levels at admission (*p* = 0.39). Low-density lipoprotein cholesterol (LDL-C) and C reactive protein (CRP) levels were slightly higher in the no-SGLT2-i group compared to the SGLT2-i group reaching a statistically significant level only in case of LDL-C (89 [58–125.8] vs. 105 [72–135] mg/dl, *p* = 0.004). CK-MB values were in physiological range in both groups without a significant elevation during the index hospitalization. Both the CK-MB levels at admission (27 [18–46] vs. 23 [16–38.8] U/l; *p* = 0.013) and maximum values (29 [21–54.5] vs. 25 [17–43] U/l; *p* = 0.009) during the index hospitalization showed significantly higher values in the SGLT2-i group in comparison to the no-SGLT2-i group.

Covariate balancing statistics and results of over-identification tests are shown in Supplementary Table 2 indicating that models adequately balanced the covariates.

### Primary and secondary outcomes

#### Infarct size and area at risk

**Fig. 2:**
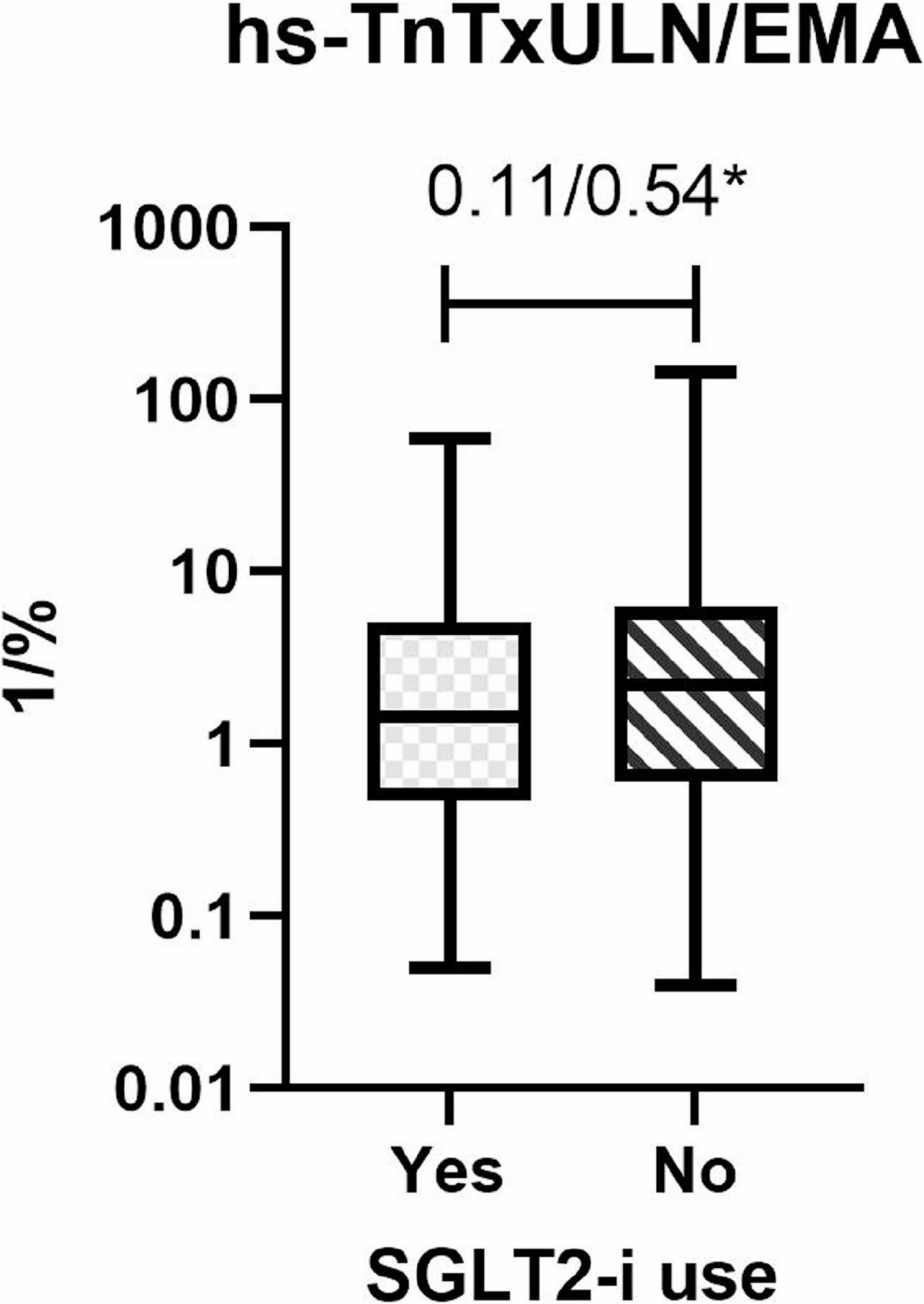
Myocardial infarction area differences between the SGLT2-i use and non-SGLT2-i use groups. Myocardial infarction area was calculated based on the coronary angiogram and maximum hs-TnT values as described in the methods section. *P-value after inverse probability weighting approach

The median number of troponin measurements in the no-SGLT2-i group was 6 [5–8], compared with 7 [6–8] in the SGLT2-i group (*p* = 0.51; Table 2). Infarct-size related parameters (both weighted and unweighted) are presented in Table 3. The maximum value of hs-TnT expressed as x ULN was numerically higher but not statistically significant in the no-SGLT2-i group compared with patients in the SGLT2-i group (55 [12.5–174] vs. 68 [22–181.6], unweighted *p* = 0.36, weighted *p* = 0.42). Patients in the SGLT2-i use group had a significantly higher EMA (41 [29–59] vs. 35 [24–59] %, unweighted *p* = 0.23, weighted *p* = 0.043). If hs-TnT values were normalized to the calculated area at risk, there was a trend favoring the SGLT2-i group (1.4 [0.47–5] vs. 2.2 [0.6–6.25], *p* = 0.11), which was not consistent after using the augmented inverse probability weighting approach (*p* = 0.54; Fig. 2). Peak hs-TnT values were not statistically different, regardless of the augmented inverse probability weighting approach.

**Table 2:** Outcomes

	SGLT2-i use				
		Yes (*N* = 105)	No (*n* = 576)	p-value	p-value after AIPW
**Characteristics of MI at presentation**					
Killip 1–2		98 (93.3)	523 (90.1)	0.39	0.26
Killip 3–4		7 (6.7)	53 (9.2)		
Life-threatening arrythmia		5 (4.8)	27 (4.7)	0.99	0.4
ST elevation		28 (26.7)	107 (18.6)	0.06	0.16
**Clinical Outcome**					
Death		4 (3.8)	24 (4.2)	0.99	0.25
Stent thrombosis		1 (1)	2 (0.4)	0.39	0.75
LVEF [%]		49.5 (43.5–55)	45 (41.8–55)	0.68	0.48
Days in hospital		4 (3–6)	4 (3–6)	0.12	0.42
Days on ICU/IMC		1 (1–1)	1 (1–2)	0.13	0.78
Recurrent myocardial infarction and hospitalisation due to heart failure		15 (2.6)	3 (2.9)	0.81	0.59
**Maximum values during the index hospitalization**					
CK-MB [U/L]		29 (21–54.5)	25 (17–43)	**0.009**	
CK [U/L]		185 (93–475)	161 (97–330)	0.30	
Creatinine [mg/dl]		1 (0.8–1.1)	1 (0.8–1.3)	0.15	
Number of hs-TnT measurements		7 (6–8)	6 (5–8)	0.51	

Continuous variables were described using median and interquartile range (IQR), categorical variables were described using n (%). p-values are from Mann–Whitney tests (continuous variables), Fisher’s exact test (categorical variables)

Bold font indicates statistical significance

*Abbreviations: AIPW *augmented inverse-probability weighting*, ICU/IMC *intensive care unit/intermediate care unit*, LVEF *left ventricular ejection fraction in the last available transthoracic echocardiogram*, SGLT2-i *Sodium-glucose Cotransporter-2 Inhibitors

**Table 3:** Myocardial infarction size

		SGLT2-i use				
			Yes (*N* = 105)	No (*n* = 576)	p-value	p-value after AIPW
Area at risk, %			41 (29–59)	35 (24–59)	0.23	**0.04**
peak hs-TnT [xULN]			55 (12.5–174)	68 (22–181.6)	0.36	0.45
peak hs-TnT [xULN/Area at risk]			1.4 (0.47–5)	2.2 (0.6–6.25)	0.11*	0.54

Continuous variables were described using median and interquartile range (IQR). *log-normal distribution, values were log-transformed, p-value is from two sided t-test. p values for non-normal distributed values are from Mann–Whitney tests without or with inverse probability weighting approach. Area at risk was calculated based on the coronary dominance, length of the LAD and localisation of the culprit lesion(s) according to the holistic coronary care algorithm using the 17-segment standardized polar map of the myocardium. The area at risk was expressed as percent (number of ischemia-affected LV segments/17*100)

*Abbreviations*: *AIPW*: augmented inverse-probability weighting; *LAD*: left anterior descending artery; *hs-TnT*: high-sensitivity cardiac troponinT; *SGLT2-i*: Sodium-glucose Cotransporter-2 Inhibitors; *TIMI*: Thrombolysis in Myocardial Infarction; *xULN*: times upper limit normal

### Clinical outcome parameters

**Fig. 3:**
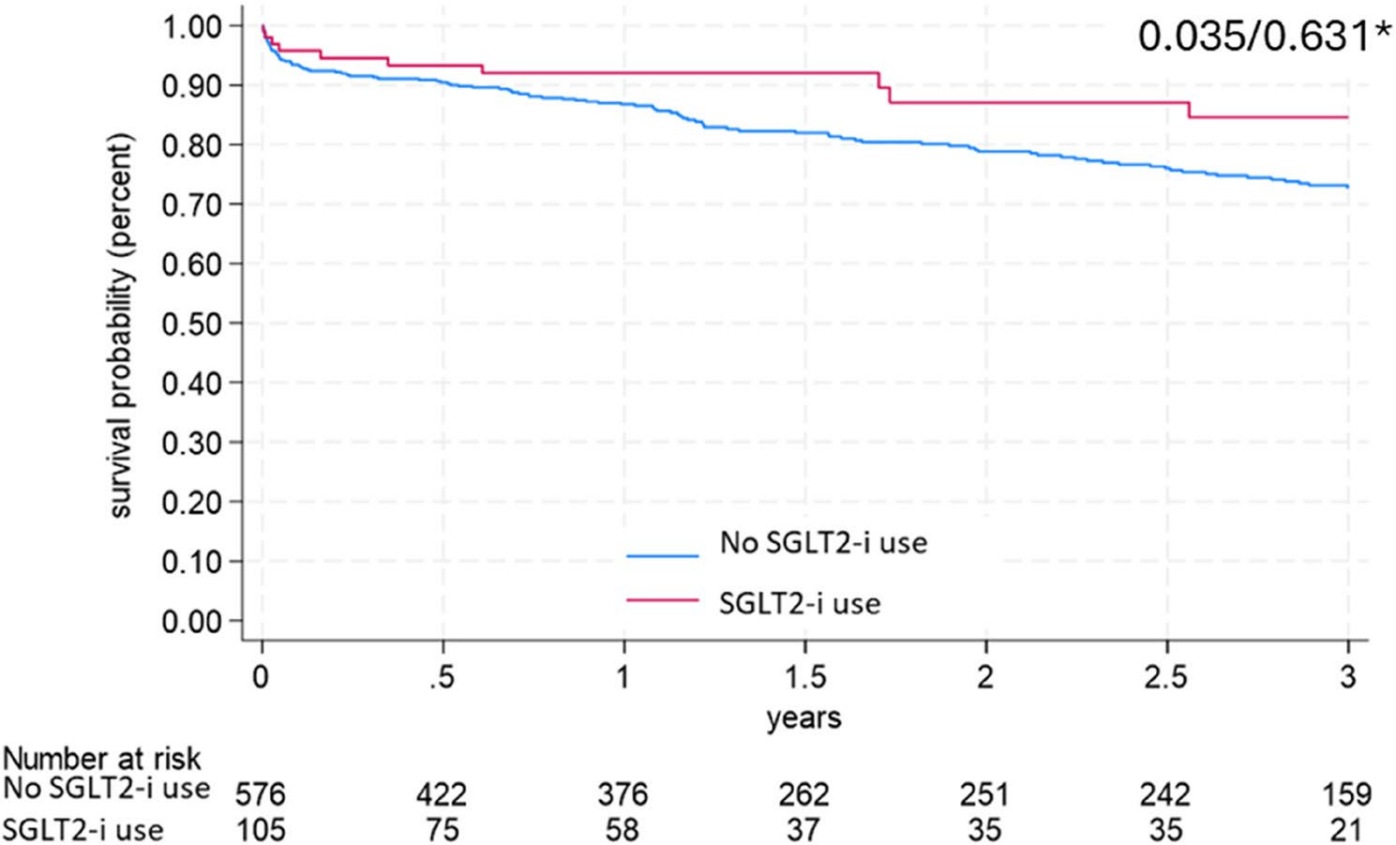
Three-year survival was analyzed using pseudo-observations and linear regression with application of the method by Overgaard, Anderson and Parmer. The overall three-year incidence of death was 25.8% and SGLT2-i therapy reduced this significantly (Log-rank test: *p* = 0.035), which, however, did not reach statistical significance after using the augmented inverse probability weighting approach (*p* = 0.926). *p-value after inverse probability weighting approach

The investigated unweighted procedural parameters are presented in Table 4. Table 2 indicates that patients were admitted to the hospital mainly in a stable condition as it is evident from the Killip classification at admission. There was a non-significant benefit associated with SGLT2-i use, regarding the Killip class upon admission (patients in Killip class 3 or 4 at admission: 6.7% vs. 9.2%, weighted *p* = 0.26). The median number of in-hospital days was 4 [3–6] in both groups. The whole duration of the in-hospital treatment was reduced by 0.4 days by patients in the SGLT2-i group compared to the no-SGLT2-i group but this difference was not statistically significant (*p* = 0.42). The median number of ICU treatment days was similar between the SGLT2-i group and the no-SGLT2-i group (1 [1–1] vs. 1 [1–2]; *p* = 0.78). Both groups had similar median ejection fraction post-MI (49.5 [43.5–55] vs. 45 [41.8–55]%; *p* = 0.48). There was 1 ischemia-related acute case of a grade 4 mitral valve insufficiency in the SGLT2-i group and none in the no-SGLT2-i group. The cumulative incidence of high-grade mitral valve insufficiency was low in both groups (2.9% vs. 0.9%, *p* = 0.11). There were no strokes in either patient groups. The in-hospital death rates were similar in both groups. (3.8% vs. 4.2%, *p* = 0.25). As an exploratory endpoint we assessed the 3-year mortality. The overall three-year incidence of death was 25.8% and SGLT2-I therapy was associated with a significantly lower mortality (Log-rank test: *p* = 0.035), which however did not reach statistical significance after using the augmented inverse probability weighting approach (*p* = 0.63) (Figure 3). The rate of recurrent myocardial infarction and hospitalization due to heart failure in the first 30 days after the index procedure was not statistically different between the two groups (2.6 vs. 2.89%, *p* = 0.589).

**Table 4:** Procedural criteria

	SGLT2-i use			
		Yes (*N* = 105)	No (*n* = 576)	p-value
Coronary arterial dominance				
Left		12 (11.9)	56 (9.7)	0.58
Right		76 (71.9)	395 (68.6)	
Intermediate		17 (16.2)	125 (21.6)	
LAD length				
Apex		62 (59)	331 (57.5)	0.34
Short		13 (12.4)	50 (8.7)	
Long		30 (28.6)	195 (33.9)	
Diseased vessels				
1		17 (15.7)	89 (15.4)	0.98
2		28 (25.9)	156 (26.9)	
3		62 (57.4)	333 (57.5)	
Acute complete occlusion		40 (38.1)	183 (31.8)	0.21
Left main intervention		11 (10.5)	59 (10.2)	0.99
Treated vessels		1 (1–2)	1 (1–2)	0.29
TIMI postinterventional				
0–2		8 (7.6)	39 (6.8)	0.68
3		97 (92.4)	537 (93.2)	
P2Y12 inhibitor used				
Ticagrelor		18 (17)	158 (27)	0.07
Prasugrel		57 (54)	260 (45)	
Clopidogrel		30 (29)	158 (27)	

Categorical variables were described using n (%). *p*-values are for Fisher’s exact test (categorical variables in 2 × 2 contingency tables) or Chi-square test (categorical variables in 2 × 3 contingency tables). Abbreviations: LAD: left anterior descending artery; hs-TnT: high-sensitivity cardiac troponin T; SGLT2-i: Sodium-glucose Cotransporter-2 Inhibitors; TIMI: Thrombolysis in Myocardial Infarction

Regarding the revascularization procedure, there was no significant difference in the use of plaque modifiers such as cutting balloon, rotablation or lithotripsy (1.9 vs. 1.56% *p* = 0.68), number of treated segments (2.2 vs. 2.1 *p* = 0.62), rate of multivessel PCI (27 vs. 33% *p* = 0.3) or PCI of chronic total occlusions (2.9 vs. 3.47% *p* > 0.99). NSTEMI patients in the SGLT2-i use group experienced a delayed PCI (> 24 h between admission and PCI) more frequently than patients in the no-SGLT2-i use group (7.2 vs. 1.5% *p* = 0.007) Fig. 3.

## Discussion

This study did not identify a relevant association between the use of SGLT2-i at the time of admission with a reduced size of MI after PCI in patients with DM.

To our current knowledge, this is the first investigation on the protective effect of an ongoing SGLT2-i therapy in diabetic patients with MI, regardless of the concomitant glucose-lowering therapy or previous CABG (coronary artery bypass graft) focusing on the anticipated and actual myocardial damage based on the coronary angiogram.

Unlike previous studies that reported potential beneficial effects, this study did not find a significant advantage of ongoing SGLT2-i therapy in case of an acute MI in patients with DM [4, 13]. However, these findings correspond to recently published, prospective, large-scale randomized trials DAPA-MI and EMPACT-MI [6, 7]. A relevant novelty of this study compared to DAPA-MI and EMPACT-MI is the inclusion of patients with relevant concomitant cardiovascular diseases. As this study did not aim to evaluate long-term cardiometabolic endpoints, a comparison in this regard is not adequate. A key innovation of this study is its integrated perspective, addressing both the comparison of peak hs-TnT levels, which indeed reflect myocardial injury size, and the potential harm caused by coronary occlusion. Hs-TnT levels are correlating with the size of MI best in case of large MI, which was given in this population according to the calculated area at risk [21]. The combination of these two parameters is highlighting the protective potential of the SGLT2-i therapy: the SGLT2-i group had a significantly more pronounced endangered myocardial area but developed a lower peak hs-TnT value. After considering the baseline characteristics of the patients and therefore addressing potential confounding factors such as the overrepresentation of heart failure in the SGLT2-i group due to the prescription indication change during the analyzed period, the SGLT2-i therapy did not prove to be associated with a reduced infarct size. The possible reduction of MI incidence under SGLT2-i therapy could not be investigated in this analysis [5]. The identified coronary dominance types and the determined area at risk is in line with observations of others [22–24].

This study also investigated major adverse in-hospital cardiovascular events, such as stroke or death, as well as the duration of hospital treatment. The 0.4% difference in mortality was not statistically significant. A well-known predictor for outcome following PCI is the Thrombolysis in Myocardial Infarction (TIMI) 3 flow in the treated segment [25]. In the present study the approx. 90% rate of achieved TIMI 3 flow after PCI in both groups signalizes a good success rate compared to others [26–29]. The in-hospital mortality in this cohort resembles mortality rates in larger referral hospitals from around the globe [26, 30].

The potential protective effects of SGLT2-i therapy in MI are diverse and not only bound to its glucosuric effects on the proximal tubule. The overall hypoglycemic effect seems to have a secondary role as well, which is best demonstrated by the fact that the glycated hemoglobin (HbA1c) level at admission was higher in the SGLT2-i using group. The worse glycemic control in this study-population compared to others could be a possible explanation of the dampened benefits of the SGLT2-i therapy [31].

A dampened inflammatory response which seems to be transmitted through NLRP3 inflammasome inhibition by Empagliflozin and Dapagliflozin may also play a relevant cardioprotective role in SGLT2-i therapy [32, 33]. Although the observed physiological leukocyte count does not point towards a systemic inflammation in either group and the CRP levels are also in the normal range, there is a marked difference between the groups regarding CRP levels favoring the SGLT2-i group as it is also evident in the work of Cesaro et al. [34]. As CRP is a well-known risk factor for cardiovascular events, a lower CRP level could also have an effect on the outcomes, as reported elsewhere [35]. The observation of the slightly reduced CRP level is in line with the study of Lannantuoni et al., who reported a direct CRP-lowering effect of Empagliflozin [36].

There was no significant difference regarding the LVEF between the two groups. This may seem contradictory to previous studies, where an SGLT2-i use did improve the LVEF after MI or heart failure with reduced ejection fraction [4, 37]. However, in this study, the initiation of the SGLT2-i therapy was before the acute event and we only had one echocardiogram during the MI-treatment, which may mask an occurred LVEF improvement. The incidence of grade 4 mitral valve insufficiency was low and only 1 case, related to the index hospitalization was observed.

In conclusion, we hypothesize that the protective effects of an ongoing SGLT2-i therapy and some advantages in the baseline characteristics such as younger age, slightly better kidney function and lower LDL-Cholesterol level in the SGLT2-i group were counterbalanced and overweighed by severe concomitant cardiovascular diseases and worse glycemic control. Upon the interpretation of our data, the change of the prescription indication during the data sampling period needs to be considered [38] that the positive study results with SGLT2-i in patients with heart failure, potentially led to the overrepresentation of heart failure patients in our cohorts with a disproportional growing tendency in the last 2 years of the analysis.

### Strengths and limitations

This trial reflcects a real-life patient cohort, accounting for several relevant concomitant diseases. Patients with severe concomitant diseases, who are mainly excluded from randomized, prospective, cardiovascular trials, such as CABG, severe renal insufficiency (GFR between 25 and 50 ml/min/1.73 m²), patients receiving insulin treatment are all represented in this trial. The results should be interpreted with some limitations. Interpatient variations in the troponin expression and concomitant diseases such as kidney failure can influence the troponin levels and therefore interfere with the infarct size estimation. There is no information about the length of SGLT2-i use or therapy adherence before the index hospitalization. There is insufficient information about diabetes-specific clinical variables such as nephropathy, neuropathy, or retinopathy. Although we aimed to control the analysis for potential confounders by using the aforementioned statistical methods, such as inverse probability weighting, this does not eliminate the biases inherent to retrospective observational studies. Moreover, the observational nature of the research will not allow to deduct causal relationships. Confounding by indication cannot be ruled out. Future studies with post-MI cardiac MRI, the golden standard of myocardial infarction-area determination [39]may deliver data that are more accurate on the size of the MI than the method used here which can only deliver an estimation of the infarct size. Whether ongoing SGLT2-i therapy reduces the incidence of MI cannot be answered with this analysis.

## Conclusion

This study did not identify a significant association between SGLT2-i therapy and MI size. Further clinical studies involving other patient populations are needed to elaborate on the possible protective effects of SGLT2-i in MI with or without diabetes mellitus.

## Supplementary Information

Supplementary Material 1.

## Data Availability

The datasets used and/or analyzed during the current study are available from the corresponding author on reasonable request.
