# Supplementary material for: SGLT2-inhibition and myocardial infarction size in patients with type 2 diabetes mellitus– Insights from an acute cardiovascular care center
Source: BMC Cardiovasc Disord. 2025 Aug 2;25:566. doi: 10.1186/s12872-025-04981-5 (PMC12317468; doi:10.1186/s12872-025-04981-5)
Supplement: Supplementary file 1 — Supplementary Material 1. [file 12872_2025_4981_MOESM1_ESM.docx]

| **Supplemental Table 1: Covariate balancing statistics** | | | | |
| --- | --- | --- | --- | --- |
| **Covariate balance summary** | | | | |
|  |  |  | Raw | Weighted |
|  | Number of obs | | 681 | 681 |
|  | Treated obs |  | 105 | 335,8 |
|  | Control obs |  | 576 | 345,2 |
|  | **Standardized**  **differences** | | **Variance ratio** | |
|  | Raw | Weighted | Raw | Weighted |
| Age | -0,43 | -0,10 | 0,97 | 0,82 |
| Sex | -0,25 | -0,04 | 0,76 | 0,97 |
| DDP4-therapy | -0,13 | 0,00 | 0,88 | 1,00 |
| Insulin-therapy | 0,13 | 0,21 | 1,04 | 1,03 |
| Diuretics-therapy | -0,09 | -0,02 | 0,97 | 0,99 |
| MRA-therapy | 0,19 | 0,01 | 1,73 | 1,02 |
| ARB-therapy | 0,20 | 0,02 | 1,19 | 1,02 |
| BB-therapy | 0,25 | -0,01 | 0,96 | 1,00 |
| Coronary heart disease | 0,29 | 0,13 | 1,01 | 1,01 |
| Heart failure | 0,27 | -0,04 | 1,75 | 0,90 |
| Smoking | -0,03 | -0,11 | 0,96 | 0,82 |
| Atrial fibrillation | -0,01 | 0,01 | 0,96 | 0,99 |
| Periperial arterial disease | -0,08 | -0,05 | 0,88 | 0,92 |
| HbA1c | 0,34 | -0,11 | 0,81 | 1,74 |
| Serum glucose at admission | -0,09 | 0,10 | 1,09 | 1,10 |
| Calcium-channel blockers | 0,05 | 0,00 | 1,05 | 1,00 |
| Ticagrelor | -0,25 | 0,07 | 0,72 | 1,07 |

*Covariate balancing statistics and results of overidentification tests are indicating that our models adequately balance the covariates. Abbreviations: ARB: angiotensin receptor blocker; BB: beta blocker; DDP4i: Dipeptidyl peptidase-4 inhibitor; MRA: mineralocorticoid receptor antagonist.*

|  | **Supplementary Table 2: Laboratory data at admission** | | | | |  |
| --- | --- | --- | --- | --- | --- | --- |
|  | | SGLT2-i use | | | | |
|  | | | Yes (N=105) | No (n=576) | p-value |  |
| Hemoglobin g/dl | | | 14.6 (13.4–15.6) | 13.3 (12–14.6) | **<0.001** |  |
| Hematocrit % | | | 43.6 (40–45.8) | 39.5 (35.8–42.8) | **<0.001** |  |
| Leucocytes 10^3^/µl | | | 9.9 (7.8–11.6) | 9.8 (7.8–12.1) | 0.79* |  |
| Platelets 10^3^/µl | | | 241 (193.5–278) | 234 (190–280) | 0.29* |  |
| Serum Creatinine mg/dl | | | 0.96 (0.8–1.15) | 1 (0.8–1.3) | 0.39 |  |
| GFR ml/min/1.73m² | | | 78 (57.5–94) | 69.8 (50–88.2) | **0.017** |  |
| HbA1C %** | | | 7.6 (6.9–8.825) | 7.2 (6.6–8) | **<0.001*** |  |
| Serum glucose mg/dl** | | | 189.5 (128-219.5) | 197.3 (143-237.8) | 0.39* |  |
| LDH U/L | | | 220 (181.5–293.5) | 224 (185–285) | 0.58* |  |
| GOT U/L | | | 31 (24–55.5) | 31 (23–51) | 0.43 |  |
| Total Serum Bilirubin mg/dl | | | 0.5 (0.3–0.6) | 0.4 (0.3–0.7) | 0.49 |  |
| TSH µIU/ml | | | 1.6 (1.1–2.4) | 1.6 (1–2.5) | 0.85 |  |
| LDL-C mg/dl | | | 89 (58–125.8) | 105 (72–135) | **0.004** |  |
| HDL-C mg/dl | | | 44 (35–51.8) | 43 (36–52) | 0.83 |  |
| CRP mg/dl | | | 0.3 (0.2–1) | 0.5 (0.2–1.5) | 0.14 |  |
| CK U/L | | | 156 (87.5–379) | 138 (87–279.8) | 0.37 |  |
| CK-MB U/L | | | 27 (18–46) | 23 (16–38.8) | **0.013** |  |
| hs-TnT xULN | | | 6.9 (2.5–26.4) | 9.5 (3.4–31.6) | 0.09 |  |

The continuous variables were described using median and interquartile range (IQR), the p-values are from Mann–Whitney tests. *Log-normal distribution, values were log-transformed, p-value is from two-sided t-test. **indicates parameters used in the covariate balancing. Abbreviations: CRP: C-Reactive Protein; CK: Creatin kinase; CK-MB: Creatin kinase-myoglobin binding; GFR: Glomerular Filtration Rate; GOT: Aspartate Aminotransferase; HbA1C: Glycated hemoglobin; HDL-C: High-density lipoprotein cholesterol; hs-TnT: high-sensitivity cardiac troponin T; LDH: Lactate Dehydrogenase; LDL-C: Low-density lipoprotein cholesterol; SGLT2-i: Sodium-glucose Cotransporter-2 Inhibitors; TSH: Thyroid-stimulating Hormone. Bold font indicates statistical significance.

Supplementary Figure 1
